# Applying Computerized Adaptive Testing to the Four-Dimensional Symptom Questionnaire (4DSQ): A Simulation Study

**DOI:** 10.2196/mental.6545

**Published:** 2017-02-21

**Authors:** Tessa Magnée, Derek P de Beurs, Berend Terluin, Peter F Verhaak

**Affiliations:** ^1^ Netherlands Institute for Health Services Research (NIVEL) Utrecht Netherlands; ^2^ EMGO Institute for Health and Care Research Department of General Practice and Elderly Care Medicine VU University Medical Center Amsterdam Netherlands; ^3^ Groningen University University Medical Center Groningen Department of General Practice Groningen Netherlands

**Keywords:** item response theory, Four-Dimensional Symptom Questionnaire, computerized adaptive testing, mental health, general practice

## Abstract

**Background:**

Efficient screening questionnaires are useful in general practice. Computerized adaptive testing (CAT) is a method to improve the efficiency of questionnaires, as only the items that are particularly informative for a certain responder are dynamically selected.

**Objective:**

The objective of this study was to test whether CAT could improve the efficiency of the Four-Dimensional Symptom Questionnaire (4DSQ), a frequently used self-report questionnaire designed to assess common psychosocial problems in general practice.

**Methods:**

A simulation study was conducted using a sample of Dutch patients visiting a general practitioner (GP) with psychological problems (n=379). Responders completed a paper-and-pencil version of the 50-item 4DSQ and a psychometric evaluation was performed to check if the data agreed with item response theory (IRT) assumptions. Next, a CAT simulation was performed for each of the four 4DSQ scales (distress, depression, anxiety, and somatization), based on the given responses as if they had been collected through CAT. The following two stopping rules were applied for the administration of items: (1) stop if measurement precision is below a predefined level, or (2) stop if more than half of the items of the subscale are administered.

**Results:**

In general, the items of each of the four scales agreed with IRT assumptions. Application of the first stopping rule reduced the length of the questionnaire by 38% (from 50 to 31 items on average). When the second stopping rule was also applied, the total number of items could be reduced by 56% (from 50 to 22 items on average).

**Conclusions:**

CAT seems useful for improving the efficiency of the 4DSQ by 56% without losing a considerable amount of measurement precision. The CAT version of the 4DSQ may be useful as part of an online assessment to investigate the severity of mental health problems of patients visiting a GP. This simulation study is the first step needed for the development a CAT version of the 4DSQ. A CAT version of the 4DSQ could be of high value for Dutch GPs since increasing numbers of patients with mental health problems are visiting the general practice. In further research, the results of a real-time CAT should be compared with the results of the administration of the full scale.

## Introduction

General practitioners (GPs) are often the first point of contact for persons with mental health problems, and they make important decisions about treatment and referrals. However, GPs vary in their ability to detect mental problems in patients during consultations [1] and may have difficulties distinguishing between “normal” psychological distress and psychopathology [2]. Moreover, time pressure in general practice is increasing.

Using a short, good quality screener to distinguish between mild psychological symptoms and severe disorders has become of particular importance for Dutch GPs, as they have been restricted to refer only patients with a Diagnostic and Statistical Manual of Mental Disorders 4th edition (DSM-IV) disorder [3] to mental health care professionals.

The Four-Dimensional Symptom Questionnaire (4DSQ; Multimedia Appendix 1) is a frequently used self-report questionnaire designed to assess common psychosocial problems in general practice [4]. It consists of four subscales measuring distress, depression, anxiety, and somatization. The 4DSQ is available in Dutch, English, and several other languages and has been widely used and validated in clinical practice. The full version of the 4DSQ comprises 50 items. It has been found that most responders need 7 minutes to complete the full version and 75% of all responders complete the 4DSQ within 10 minutes [4]. Responses to the questionnaire can be used to distinguish between patients with “normal” psychological distress and patients with psychopathology [5-7]. This is of increasing importance for GPs who have to make crucial decisions about the triage of patients with mental health problems.

Computerized adaptive testing (CAT) is a method to reduce patient burden of traditional questionnaires, by letting a computer dynamically select only the items that give new information about the patient. Based on a patient’s answer to a single first item, a responders underlying trait (eg, level of depression) is estimated. In addition, an automated algorithm selects the next item that is most appropriate or informative for this responder. The benefit of using CAT is the reduction in items without a loss in reliability or precision in measurement [8].

CAT relies on item response theory (IRT) [9]. A CAT version of the Center for Epidemiologic Studies-Depression (CES-D) scale, one of the most widely used depression screeners, provided only marginally different outcomes with a decreased number of items compared to the full version [10]. CAT has also been applied successfully to other mental health questionnaires, such as the Beck Depression Inventory [11], the Beck Scale for Suicide Ideation [12], and the 90-item Mood and Anxiety Symptom Questionnaire [13] and seems more accurate than a simple short-form version of an assessment [14]. It is not clear yet if the efficiency of screening for common mental health problems in general practice can be increased by developing an adaptive version of the 4DSQ.

The aims of this simulation study were (1) to investigate if responses of a clinical sample to a paper-and-pencil version of the 4DSQ meet the psychometric requirements needed for IRT; and (2) to determine if a simulated adaptive version of the 4DSQ would yield inferences similar to those based on the full version of the 4DSQ. This simulation study is the first step necessary for the development of a CAT version of the 4DSQ.

## Methods

### Participants

We used data collected in the baseline measurement of a study evaluating triage decisions in general practice. All patients with mental health problems visiting a GP working in a primary care center in the northern part of the Netherlands between January 1 and December 31, 2014 were included in the study (N=408). All included participants provided informed consent. Participants filled in the Dutch paper-and-pen version of the 4DSQ and only patients with complete data were included in the analyses (92.9%, 379/408). As a result, our final sample consisted of 379 participants with a mean age of 44.8 years (SD 16.5, range 16 to 87). Of the participants, 66.8% (253/379) were female. No significant differences in age (*P*=.715) or sex (*P*=.205) were found between responders with complete and without complete data.

### Psychometric Evaluation

Since all four of the 4DSQ scales are used and interpreted separately, we performed the psychometric evaluation and our analyses for each of the four scales separately. We followed the five steps described in the analysis plan used for the PROMIS study, which was aimed at improving patient-reported outcome instruments [8].

#### Step 1: Descriptive Statistics

Descriptive statistics were calculated for each single item (Multimedia Appendix 2). The 4DSQ consists of questions about complaints and symptoms that occurred during the previous week, such as “During the past week, did you feel tense?” Responders indicated how often they experienced these symptoms by answering “no,” “sometimes,” “regularly,” “often,” or “very often or constantly.” According to the scoring protocol, responses were coded as 0 (no), 1 (sometimes), 2 (regularly, often, or very often/constantly). The four 4DSQ scales vary in the total number of items: 16 items for distress, 6 for depression, 12 for anxiety, and 16 for somatization. A total score was calculated for each scale by adding up all item scores. To examine internal consistency, Cronbach alpha was calculated for each scale, with .8 as the acceptable minimum. We analyzed whether removing any of the items changed the internal consistency of a scale.

#### Step 2: Evaluate Item Response Theory Assumptions

Within IRT, data have to agree with three basic assumptions: unidimensionality, local independency, and monotonicity [8].

Unidimensionality means that a person’s response to an item is accounted for by his or her level on the underlying trait and not by any other factor. A confirmatory factor analysis (CFA) with ordinal data was performed to study unidimensionality for each scale. The model’s fit was assessed using four frequently used fit indices: comparative fit index (CFI) greater than 0.95 for good fit, root mean square error of approximation (RMSEA) less than 0.06 for good fit, Tucker Lewis index (TLI) greater than 0.95 for good fit, and standardized root mean residuals (SRMR) less than 0.08 for good fit.

Local independence means that there should be no significant association among item responses, except for the association controlled for by the underlying trait. This assumption was checked by inspecting residual correlations between item pairs within the CFA. Items with high residual correlations (greater than 0.2) were considered as possibly locally dependent.

The assumption of monotonicity means that an item response related to a higher level of the trait should increase with the level of the trait. This assumption was studied by plotting trace lines. In addition, we studied scalability coefficients of IRT probability curves (greater than 0.3 indicates monotonicity).

#### Step 3: Graded Response Model Fit

Within IRT, several models are commonly used; however, because of the ordered-response categories of the 4DSQ, a graded response model (GRM) was preferred for our data [15]. This model estimates at which levels of an underlying trait (θ), such as depression, a person is likely to choose one of the response options of an item. For each single item, several GRM parameters are estimated. The discrimination parameter (α) represents the extent to which an item discriminates between different trait levels. An item with a high alpha is strongly associated with the measured construct. Two difficulty or threshold parameters (ß_1_ and ß_2_) were also estimated. A category response curve (CRC), based on the estimated parameters, was plotted for each item to evaluate the fit of the model to the data.

#### Step 4: Differential Item Functioning

An item displays differential item functioning (DIF) if persons with different characteristics (eg, males and females) respond differently to an item, despite equivalent levels of the underlying trait [8]. Items showing DIF may bias CAT outcomes. To check for DIF (uniform and non-uniform), GRM estimates of each item were compared between subgroups varying in gender (male or female) and age (R^2^ less than .03 indicating no DIF).

#### Step 5: Simulated Computerized Adaptive Testing

The GRM parameter estimates from Step 3 were used for a CAT simulation. As no information on a subject is available before the first item is administered, θ is initially set at 0. After the first item is answered, the choice for the next item is based on the GRM parameters of all potential next items in relation to the response to the item that was answered first. All optimal next items are selected based on the maximum Fisher estimation method. The CAT selects new items until a pre-defined stopping rule is reached. A stopping rule is based on either a maximum number of items administered or on a pre-specified level of measurement precision [10-13].

We combined the two following stopping rules: (1) stop when the standard error of the trait is similar to the standard error of the full lengths scale, or (2) stop when half the number of the full scale is administered. We compared CAT outcomes with the first stopping rule only and with both stopping rules. Regarding the first stopping rule, we inspected varying levels of standard error (from 0.2 to 0.8). The pre-defined standard error of theta that corresponded with the standard error of the full scale was used as a reference point. Correlations were calculated between trait levels based on CAT and on the scores from the full version of the 4DSQ. We added a second stopping rule because questionnaires in mental health often are most informative for patients with relatively high levels of clinical outcomes [10,16,17]. For patients with a low level of the assessed outcome (eg, patients with low levels of depression), many items provide little (additional) information. Ironically, as the CAT algorithm finds it difficult to estimate the standard error when items offer little information, patients with a low trait level often have to answer all items, even though they provide no new information.

### Software

The descriptive statistics and the estimation of the GRM parameters were done in STATA 14.0. The CFA model was estimated using the lavaan package in R [18,19]. Monotonicity was checked using the R mokken package [20] and DIF with the R lordif package [21]. The CAT simulation was done with the CatIRT package in R [22].

## Results

### Step 1: Descriptive Statistics

The sample’s mean total score on the 4DSQ distress scale was 18.6 (SE 0.43, range 0-32, median 20), with an overall Cronbach alpha of .92. The mean depression score was 3.4 (SE 0.20, range 0-12, median 2), with a Cronbach alpha of .90. The mean score for anxiety was 5.5 (SE 0.27, range 0-23, median 4), with a Cronbach alpha of .87. Finally, for the somatization scale, the sample scored 11.6 on average (SE 0.35, range 0-32, median 11), with a Cronbach alpha of .85. These results were comparable to other studies [4,7]. The descriptive statistics of the single items on the four scales are shown in Multimedia Appendix 2. Removing any one of the items did not change the internal consistency of any of the four scales.

### Step 2: Checking Item Response Theory Assumptions

Regarding the first assumption, unidimensionality, we concluded that the items of the anxiety scale showed a good model fit for all four fit indices of the CFA. The items of the distress and depression scales showed a good fit for three of the four indices, but not for RMSEA, although they nearly did. For good fit, RMSEA should be lower than 0.06, but it was 0.08 (distress) and 0.07 (depression). The items of the somatization scale showed good fit for two out of four indices, but not for RMSEA (0.07 instead of less than 0.06) and TLI (0.94 instead of greater than 0.95).

Regarding the second assumption, out of 321 items pairs within the four scales (equation 1), two item pairs with a residual correlation above 0.2 were observed, indicating local independency. They were items 20 and 39 (sleep-related), and items 47 and 48 (trauma-related), all from the distress scale.

321=(½)(6)(5) + (½)(16)(15) + (½)(12)(11) + (½)(16)(15) (1)

The scalability coefficient of all items was higher than 0.3, indicating that all items met the third assumption of monotonicity.

### Step 3: Graded Response Model Fit

The parameter estimates of the GRM for all items of the four scales are shown in Multimedia Appendix 3. Item 33 (“would be better off dead”) of the depression scale showed the highest alpha (7.377) and discriminates best between persons with low and high levels of depression. For the three other scales, the highest alphas were observed for item 37 (3.483, distress, “no longer feel like doing anything”), item 27 (5.527, anxiety, “feel frightened”), and item 16 (1.855, somatization, “pain in the chest”). All other items showed an alpha above 1, except for items 47 and 48 (distress), item 50 (anxiety), and items 6 and 8 (somatization).

It was found that 43 items showed CRCs as expected. Five items on the anxiety scale (40, 42, 43, 49, and 50) and two items on the somatization scale (5 and 14) did not show CRCs as expected. For those items, the probability to answer “sometimes” was always lower than the probability for one of the other responses, regardless of the trait level.

**Figure 1:**
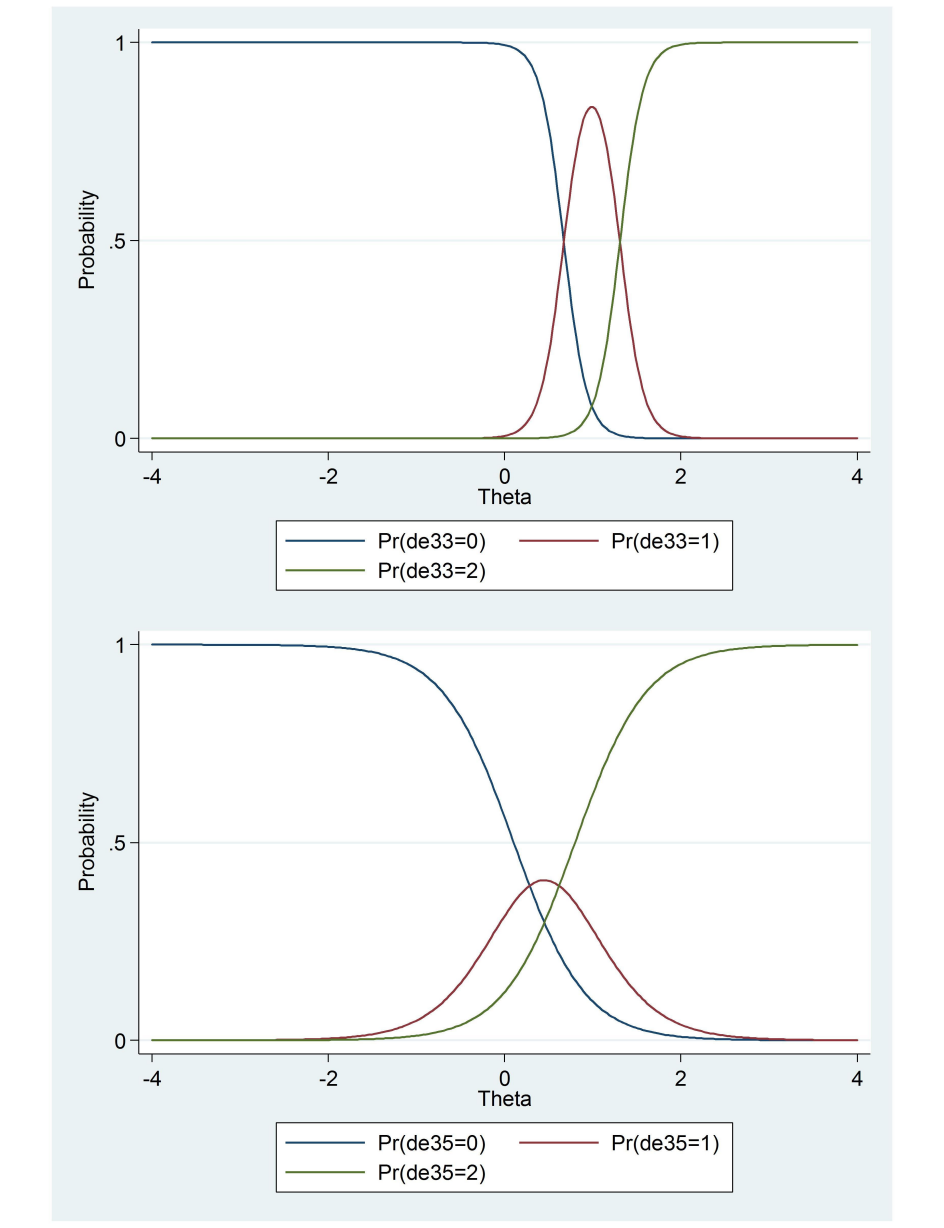
Category response curves of items 33 and 35 of the Four-Dimensional Symptom Questionnaire depression scale. The probability (y-axis) represents the chance on a certain response (0=never; 1=sometimes; 2=regularly, often, very often, or constantly) given a certain level of theta. Theta (x-axis) represents the underlying trait level; in this figure, depression. The abbreviation Pr is probability.

As an example, Figure 1 shows the CRCs of the items with the highest (item 33; α=7.377, ß_1_=0.688, ß_2_=1.349) and lowest (item 35; α=2.457, ß_1_=0.119, ß_2_=0.828) discrimination parameter (α) of the depression scale. The higher discrimination parameter of item 33 indicates an ability to demarcate fine gradations between persons with similar levels of depression. This can be observed in Figure 1, which shows steep curves for different answer categories for item 33. Item 35 (no escape from situation) is more easily endorsed than item 33 in general (would be better off dead), which is indicated by the location of the curves more on the left side of the graph. Persons with a high depression level are most likely to answer “sometimes” to item 33, and to answer “regularly”, “often,” or “very often or constantly” to item 35.

### Step 4: Differential Item Functioning

For the depression, anxiety, and somatization subscales, no items showed DIF. The only item that showed significant and relevant uniform and non-uniform DIF was item 41 (“I quickly get emotional”) from the distress scale for the covariate gender.

### Step 5: Simulated Computerized Adaptive Testing

The characteristics of the simulated CAT under different levels of measurement precision (allowing the standard error of the estimated underlying trait to gradually increase; stopping rule 1) are shown in Table 1. For each scale, the standard error of theta that was equal to the standard error of the full version scale is indicated. For example, the standard error of the full version scale of distress was 0.4. When allowing the standard error of theta to be maximal 0.4, the mean number of items administered could be decreased from 16 to 6.3. The correlation between the distress level based on 6.3 items and the distress level based on all items was high (0.96). Comparable results were found for the three other scales. With the first stopping rule, we were able to reduce the mean number of items administered to 5 for depression (from 6), to 8.3 for anxiety (from 12), and to 12.9 for somatization (from 16), while correlations between CAT and full test scores remained high. Applying CAT with the first stopping rule to all four scales could reduce the total number of 4DSQ items from 50 to, on average, 34 items.

**Table 1:** Mean number of items administered under varying levels of measurement precision and correlations between computerized adaptive testing scores and full version scores of the Four-Dimensional Symptom Questionnaire.

Stopping rule	Distress		Depression		Anxiety		Somatization	
	Number of items, mean (SD)	Correlation^a^	Number of items, mean (SD)	Correlation^a^	Number of items, mean (SD)	Correlation^a^	Number of items, mean (SD)	Correlation^a^
None	16	1.00	6	1.00	12	1.00	16	1.00
SE^b^ (θ)<0.2	15.7 (0.8)	1	5.7 (0.9)^c^	1^c^	12 (0)	1	16 (0)	1
SE (θ)<0.3	8.8 (4.5)	0.98	5.4 (1.2)	0.99	8.7 (4.3)^c^	0.97^c^	14 (0)	0.97
SE (θ)<0.4	6.3 (4.3)^c^	0.96^c^	5.0 (1.3)	0.99	8.3 (4.3)	0.97	12.9 (2.1)^c^	0.95^c^
SE (θ)<0.5	4.9 (3.8)	0.92	4.9 (1.4)	0.99	8.1 (4.4)	0.97	11.2 (4.9)	0.95
SE (θ)<0.6	4.1 (2.6)	0.86	4.6 (1.4)	0.99	5.9 (4.2)	0.94	7.5 (4.6)	0.86
SE (θ)<0.7	3.8 (2.5)	0.84	3.9 (1.3)	0.97	5.9 (4.1)	0.94	4.6 (3.4)	0.73
SE (θ)<0.8	3.7 (2.3)	0.79	3.9 (1.3)	0.97	5.6 (4.0)	0.93	4.6 (3.4)	0.73

^a^Correlation between CAT θ and complete test θ.

^b^SE: standard error.

^c^The standard error of theta (θ) is equal to the standard error of the full version scale.

The results of combining the first stopping rule with the second stopping rule are shown in Table 2. For distress, the average number of items could be further decreased from 6.3 to 5, but the correlation also decreased from 0.96 to 0.79. Therefore, we did not apply the second stopping rule to this scale. For the three other scales, the number of average items could be decreased, while the correlation remained high. Overall, when applying the CAT with both stopping rules (except for distress), the 4DSQ could be reduced from 50 to 22 items.

**Table 2:** Mean number of items administered and correlation with total estimated theta under one or two stopping rules.

Stopping rule	Distress		Depression		Anxiety		Somatization	
	Number of items, mean (SD)	Correlation^a^	Number of items, mean (SD)	Correlation^a^	Number of items, mean (SD)	Correlation^a^	Number of items, mean (SD)	Correlation^a^
None	16	1.00	6	1.00	12	1.00	16	1.00
SE^b^ (θ) = SE (full)	6.3 (4.3)	0.96	5.4 (1.2)	0.99	8.7 (4.3)	0.97	12.9 (2.1)	0.95
Maximum items^c^	5.0 (2.1)	0.79	3.0 (0)	0.96	4.9 (1.4)	0.92	7.9 (0.3)	0.92

^a^Correlation between CAT θ and complete test θ.

^b^SE: standard error.

^c^Maximum items are determined by dividing the number of items by 2.

## Discussion

### Principal Findings

In summary, when applying CAT to the 4DSQ and applying two stopping rules to the subscales of anxiety, depression, somatization, and one stopping rule to the subscale distress, the total number of items on the 4DSQ could be reduced by 56% on average (from 50 to 22 items), without losing a considerable amount of measurement precision.

### Interpretation

Our simulation study showed that CAT may increase the efficiency of the 4DSQ and could reduce responders’ burden by more than 50%. These results were also found in other CAT studies, such as on the *Center for Epidemiological Studies-Depression Scale* (CES-D), where the total scale of 20 items could be reduced to 7 items [23].

Some CATs to measure anxiety and depression have already been used and evaluated in clinical (specialist) care [24-26]. These CATs appeared to be useful for longitudinal monitoring of symptoms, since they were as reliable over time as traditional questionnaires [27].

A CAT version of the 4DSQ seems especially useful in general practices, for example, as part of a broad online assessment to investigate the severity of psychological problems of patients. As the number of patients visiting their GP with mental health problems is increasing [28], there is a growing need for an efficient screener for mental health problems. Many Dutch GPs already use the 4DSQ. An efficient, shortened 4DSQ could be combined with other mental health questionnaires, while keeping responders’ burden as low as possible. GPs have only a limited time and often have to make important decisions about referring patients with mental health problems. An online severity assessment, ideally preceding the first consultation, could be helpful as a first quick evaluation on which to base further (treatment) decisions. Some GPs use the 4DSQ as an agenda-setting tool to talk about the psychological problems of their patients. An online assessment could fulfill the same agenda-setting function.

However, some obstacles for the successful implementation of a CAT version of the 4DSQ in general practice exist. First, current information and communication technology (ICT) possibilities in general practices are insufficient for the implementation of CAT, which requires sophisticated statistical software. Second, it is not clear to what extent GPs are willing to implement a CAT version of the 4DSQ. GPs may use responses from individual 4DSQ items, such as item 47 or 48 on traumatic events, for a quick clinical evaluation, and this information may be lost when applying CAT. Lastly, it is not clear if CAT is appropriate for all patients. Previous research on CAT after inpatient rehabilitation suggests that it might only be feasible to collect (complete) data for a specific subset of patients [29]. Some patients may prefer a paper-and-pencil version of a questionnaire to an online assessment. Although a CAT version of the 4DSQ might not be immediately available for use in clinical practice, some studies have already shown that CAT versions of traditional questionnaires can be used in a clinical setting [24-26] and are well accepted by patients [25]. Recently developed, free-to-use online CAT platforms [30,31] are likely to enable the development of new CAT questionnaires. Moreover, some Dutch GPs already have been using an online screener to assess mental health problems, so application of a CAT version of the 4DSQ in clinical practice may be within reach.

### Strengths and Limitations

As this was a simulation study, we used responses to a paper-and-pencil version of the 4DSQ. In reality, responders might behave differently when receiving a computerized adaptive assessment. For example, we do not know if the actual computer administration might influence responses or what effect differences in the item order may have. However, a previous study showed that differences between results from a simulation CAT and a real CAT were small [32]. We used data from a sample from a northern region of the Netherlands, but parameter estimates based on data from different regions and countries might also differ.

Regarding the psychometric evaluation, our data showed some weaknesses. For most items of the four subscales of the 4DSQ, the assumptions for an IRT analysis were met. The assumption of unidimensionality was not met perfectly for all four scales, although it nearly was. Moreover, some items showed other limitations, such as correlations between item pairs or differential item functioning. These items might be left out in future (real-time) CAT versions of the 4DSQ. As in other studies, we found relevant DIF for the item “emotionality” on the distress scale. Women tend to more easily agree with this item compared to men, even when they have a similar underlying level of distress. When looking at the individual responses to the CAT of the distress scale, the item “emotionality” was only administered to participants with a very low level of distress. This indicates that the DIF on this item does not bias the CAT outcomes, as this item is not informative enough to be included in the final CAT. When looking at the distribution and the CRC of some items of the anxiety and somatization scales, participants either endorse option 0 or option 1 to 2. Patients apparently have difficulties differentiating between response categories 1 and 2. This might be solved in future studies by grouping response options 1 and 2 for certain items, making them dichotomous.

### Conclusions

Data from this simulation study in general agreed with assumptions needed for CAT. CAT seems useful for improving the efficiency of the 4DSQ by 56%, without losing a considerable amount of measurement precision. Of course, this simulation study is only the first step towards a CAT version of the 4DSQ that could be implemented in clinical practice and it should be followed by a study on a real-time CAT and eventually by an evaluation of the developed CAT version in a clinical setting.

## Appendix

Multimedia Appendix 1 English version of the Four-Dimensional Symptom Questionnaire.

Multimedia Appendix 2 Descriptive statistics of items of the Four-Dimensional Symptom Questionnaire.

Multimedia Appendix 3 Graded response model parameter estimates of the Four-Dimensional Symptom Questionnaire.

## Abbreviations

4DSQ: Four-Dimensional Symptom Questionnaire
CAT: computerized adaptive testing
CRC: category response curve
CFA: confirmatory factor analysis
DIF: differential item functioning
GP: general practitioner
GRM: graded response model
IRT: item response theory
RMSEA: root mean square error of approximation
