# Supplementary material for: Applying Computerized Adaptive Testing to the Four-Dimensional Symptom Questionnaire (4DSQ): A Simulation Study
Source: JMIR Ment Health. 2017 Feb 21;4(1):e7. doi: 10.2196/mental.6545 (PMC5340924; doi:10.2196/mental.6545)
Supplement: Multimedia Appendix 1 [file mental_v4i1e7_app1.pdf]

Four-Dimensional Symptom Questionnaire (4DSQ)

The following is a list of questions about various complaints and symptoms you may have. Each question refers to the complaints and symptoms that you had **in the past week (the past 7 days, including today)**. Complaints you had before then, but no longer had during the past week, do not count.
Please indicate for each complaint how often you noticed that you had it in the past week by putting an “X” in the box under the answer that is most appropriate.

During the past week, did you suffer from:

		no	sometimes	regularly	often	very often or constantly
1.	dizziness or feeling light-headed?	☐	☐	☐	☐	☐
2.	painful muscles?	☐	☐	☐	☐	☐
3.	fainting?	☐	☐	☐	☐	☐
4.	neck pain?	☐	☐	☐	☐	☐
5.	back pain?	☐	☐	☐	☐	☐
6.	excessive sweating?	☐	☐	☐	☐	☐
7.	palpitations?	☐	☐	☐	☐	☐
8.	headache?	☐	☐	☐	☐	☐
9.	a bloated feeling in the abdomen?	☐	☐	☐	☐	☐
10.	blurred vision or spots in front of your eyes?	☐	☐	☐	☐	☐
11.	shortness of breath?	☐	☐	☐	☐	☐
12.	nausea or an upset stomach?	☐	☐	☐	☐	☐

During the past week, did you suffer from:

		no	sometimes	regularly	often	very often or constantly
13.	pain in the abdomen or stomach area?	☐	☐	☐	☐	☐
14.	tingling in the fingers?	☐	☐	☐	☐	☐
15.	pressure or a tight feeling in the chest?	☐	☐	☐	☐	☐
16.	pain in the chest?	☐	☐	☐	☐	☐
17.	feeling down or depressed?	☐	☐	☐	☐	☐
18.	sudden fright for no reason?	☐	☐	☐	☐	☐
19.	worry?	☐	☐	☐	☐	☐
20.	disturbed sleep?	☐	☐	☐	☐	☐
21.	a vague feeling of fear?	☐	☐	☐	☐	☐
22.	lack of energy?	☐	☐	☐	☐	☐
23.	trembling when with other people?	☐	☐	☐	☐	☐
24.	anxiety or panic attacks?	☐	☐	☐	☐	☐

During the past week, did you feel:

		no	sometimes	regularly	often	very often or constantly
25.	tense?	☐	☐	☐	☐	☐
26.	easily irritated?	☐	☐	☐	☐	☐
27.	frightened?	☐	☐	☐	☐	☐

© Copyright 1996 by Dr. B. Terluin, Almere, the Netherlands, text revision 2010

	no	sometimes	regularly	often	very often or constantly
**During the past week, did you feel:**					
28. that everything is meaningless? - - - - - - - - - - - - - -	☐	☐	☐	☐	☐
29. that you just can't do anything anymore? - - - - - - -	☐	☐	☐	☐	☐
30. that life is not worth while? - - - - - - - - - - - - - - - - -	☐	☐	☐	☐	☐
31. that you can no longer take any interest in the people and things around you? - - - - - - - - - - - - - -	☐	☐	☐	☐	☐
32. that you can't cope anymore? - - - - - - - - - - - - - -	☐	☐	☐	☐	☐
33. that you would be better off if you were dead? - - -	☐	☐	☐	☐	☐
34. that you can't enjoy anything anymore? - - -- - - - -	☐	☐	☐	☐	☐
35. that there is no escape from your situation? - - - - -	☐	☐	☐	☐	☐
36. that you can't face it anymore? - - - - - - - - - - - - -	☐	☐	☐	☐	☐
**During the past week, did you:**					
37. no longer feel like doing anything? - - - - - - - - - - -	☐	☐	☐	☐	☐
38. have difficulty in thinking clearly? - - - - - - - - - - - -	☐	☐	☐	☐	☐
39. have difficulty in getting to sleep? - - - - - - - - - - - -	☐	☐	☐	☐	☐
40. have any fear of going out of the house alone? - - -	☐	☐	☐	☐	☐
**During the past week:**					
41. did you easily become emotional? - - - - - - - - - - -	☐	☐	☐	☐	☐
42. were you afraid of anything when there was really no need for you to be afraid? - - - - - - - - - - - - - *(for instance animals, heights, small rooms)*	☐	☐	☐	☐	☐
43. were you afraid to travel on buses, streetcars/ trams, subways or trains? - - - - - - - - - - -- - - - - - -	☐	☐	☐	☐	☐
44. were you afraid of becoming embarrassed when with other people? -	☐	☐	☐	☐	☐
45. did you ever feel as if you were being threatened by unknown danger? -	☐	☐	☐	☐	☐
46. did you ever think "I wish I was dead"? - - - - - - - -	☐	☐	☐	☐	☐
47. did you ever have fleeting images of any upsetting event(s) that you have experienced? - - - - - - - - -	☐	☐	☐	☐	☐
48. did you ever have to do your best to put aside thoughts about any upsetting event(s)? - - - - - - - -	☐	☐	☐	☐	☐
49. did you have to avoid certain places because they frightened you? -	☐	☐	☐	☐	☐
50. did you have to repeat some actions a number of times before you could do something else? - - - - -	☐	☐	☐	☐	☐

© Copyright 1996 by Dr. B. Terluin, Almere, the Netherlands, text revision 2010

4DSQ scoring form

'no' = score 0
'sometimes' = score 1
'regularly' or more often = score 2

Distress	Depression	Anxiety	Somatisation
17	28	18	1
19	30	21	2
20	33	23	3
22	34	24	4
25	35	27	5
26	46	40	6
29		42	7
31		43	8
32		44	9
36		45	10
37		49	11
38		50	12
39			13
41			14
47			15
48			16

Interpretation:

	Distress	Depression	Anxiety	Somatisation
moderately elevated:	> 10	> 2	> 3	> 10
strongly elevated:	> 20	> 5	> 8*	> 20

* cut-off point is adjusted to compensate for differential item functioning

The 4DSQ is copyright protected work. The copyright owner is Dr. B. Terluin. The 4DSQ is free for non-commercial use as in (mental) health care and scientific research. Commercial use of the 4DSQ requires a licence agreement. More information, translations and literature references can be found at www.emgo.nl/researchtools/4dsq.asp.

Validation status of the English translation of the 4DSQ

Validated, published,
with adjustment of the higher cut-off point of the anxiety scale

The English translation was validated in a Canadian sample of family medicine attendees and their data were compared with a Dutch sample of general practice attendees using confirmatory factor analysis (CFA) and differential item functioning (DIF) analysis. The English scales were as reliable as the Dutch scales (Cronbach's alphas between 0.82 and 0.92) and had the same validity as the Dutch scales. DIF was present in 7 items but impacted only the anxiety sum score. Lowering the higher cut-off for anxiety ensures that the cut-off retains the same meaning (with respect to ruling in anxiety disorder) as the Dutch cut-off. The English distress, depression and somatization scales measure the same constructs as the Dutch scales and use the same cut-off points.

Reference:
Terluin B, Smits N, Miedema B. The English version of the four-dimensional symptom questionnaire (4DSQ) measures the same as the original Dutch questionnaire: A validation study. Eur J Gen Pract 2014; Apr 29 [Epub ahead of print].

June 28, 2014
