# Supplementary material for: Applying Computerized Adaptive Testing to the Four-Dimensional Symptom Questionnaire (4DSQ): A Simulation Study
Source: JMIR Ment Health. 2017 Feb 21;4(1):e7. doi: 10.2196/mental.6545 (PMC5340924; doi:10.2196/mental.6545)
Supplement: Multimedia Appendix 2 [file mental_v4i1e7_app2.pdf]

Table 1A. Descriptive statistics of single items on the 4DSQ distress scale

Item #	Item	Answer category			Mean (SD)	Cronbach's α after removing this item
		0	1	2		
17	Feeling down or depressed	84	113	183	1.26 (0.80)	0.91
19	Worry	31	78	271	1.63 (0.63)	0.92
20	Disturbed sleep	66	100	214	1.39 (0.77)	0.91
22	Lack of energy	80	106	194	1.30 (0.80)	0.91
25	Tense	43	90	247	1.54 (0.69)	0.91
26	Easily irritated	75	117	188	1.30 (0.78)	0.91
29	Can't do anything anymore	117	117	146	1.08 (0.83)	0.91
31	No longer any interest	167	114	99	0.82 (0.82)	0.91
32	Can't cope anymore	125	127	128	1.01 (0.82)	0.91
36	Can't face it anymore	135	126	119	0.96 (0.82)	0.91
37	No longer feel like doing anything	106	134	140	1.09 (0.80)	0.91
38	Have difficulty in thinking clearly	112	131	137	1.07 (0.81)	0.91
39	Have difficulty in getting to sleep	116	112	152	1.09 (0.84)	0.92
41	Easily become emotional	84	114	182	1.26 (0.80)	0.91
47	Fleeting images of upsetting event(s)	134	135	111	0.94 (0.80)	0.92
48	Do your best to put aside thought about upsetting event(s)	157	112	111	0.88 (0.83)	0.92

Table 1B. Descriptive statistics of single items on the 4DSQ depression scale

Item #	Item	Answer category			Mean (SD)	Cronbach's α after removing this item
		0	1	2		
28	Everything meaningless	186	97	97	0.77 (0.83)	0.88
30	Life is not worth while	237	80	63	0.54 (0.76)	0.87
33	Would be better off dead	290	59	31	0.32 (0.62)	0.88
34	Can't enjoy anything anymore	165	114	101	0.83 (0.82)	0.89
35	No escape from situation	205	84	91	0.70 (0.83)	0.89
46	Think I wish I were dead	300	53	27	0.28 (0.59)	0.88

Table 1C. Descriptive statistics of single items on the 4DSQ anxiety scale

Item #	Item	Answer category			Mean (SD)	Cronbach's α after removing this item
		0	1	2		
18	Sudden fright for no reason	233	84	63	0.55 (0.76)	0.87
21	Vague feeling of fear	146	100	134	0.97 (0.86)	0.86
23	Trembling when with other people	278	61	41	0.38 (0.67)	0.86
24	Anxiety or panic attacks	249	80	51	0.48 (0.72)	0.86
27	Frightened	168	106	106	0.84 (0.83)	0.85
40	Fear of going out of the house alone	294	46	40	0.33 (0.66)	0.86
42	Afraid of anything no need for	277	46	57	0.42 (0.74)	0.87
43	Afraid to travel on buses or other	320	35	25	0.22 (0.55)	0.87
44	Afraid of becoming embarrassed when with other people	233	97	50	0.52 (0.72)	0.87
45	Threatened by unknown danger	305	46	29	0.27 (0.59)	0.86
49	Avoid certain places because they frightened you	313	36	31	0.26 (0.59)	0.87
50	Repeat some actions a number of times	304	47	29	0.28 (0.59)	0.87

Table 1D. Descriptive statistics of single items on the 4DSQ somatization scale

Item #	Item	Answer category			Mean (SD)	Cronbach's α after removing this item
		0	1	2		
1	Dizziness or feeling light-headed	115	169	96	0.95 (0.74)	0.84
2	Painful muscles	130	98	152	1.06 (0.86)	0.84
3	Fainting	364	15	1	0.04 (0.22)	0.85
4	Neck pain	156	102	122	0.91 (0.85)	0.84
5	Back pain	157	91	132	0.93 (0.87)	0.84
6	Excessive sweating	164	113	103	0.84 (0.82)	0.85
7	Palpitations	179	116	85	0.75 (0.80)	0.84
8	Headache	90	129	161	1.19 (0.79)	0.85
9	A bloated feeling in the abdomen	178	115	87	0.76 (0.80)	0.84
10	Blurred vision or spots in front of eyes	186	114	80	0.72 (0.79)	0.84
11	Shortness of breath	241	83	56	0.51 (0.74)	0.84
12	Nausea or an upset stomach	172	121	87	0.78 (0.80)	0.84
13	Pain in the abdomen or stomach area	199	104	77	0.68 (0.79)	0.84
14	Tingling in the fingers	257	70	53	0.46 (0.73)	0.84
15	Pressure of tight feeling in the chest	206	101	73	0.65 (0.78)	0.84
16	Pain in the chest	278	68	34	0.36 (0.64)	0.84
