# Supplementary material for: Applying Computerized Adaptive Testing to the Four-Dimensional Symptom Questionnaire (4DSQ): A Simulation Study
Source: JMIR Ment Health. 2017 Feb 21;4(1):e7. doi: 10.2196/mental.6545 (PMC5340924; doi:10.2196/mental.6545)
Supplement: Multimedia Appendix 3 [file mental_v4i1e7_app3.pdf]

Table 1A. Estimated GRM parameters of the 4DSQ distress scale items

Item #	Item	α	(SE)	$ß_1$	(SE)	$ß_2$	(SE)
17	Feeling down or depressed	2.516	0.254	-0.935	0.096	0.046	0.076
19	Worry	2.367	0.273	-1.736	0.150	-0.702	0.088
20	Disturbed sleep	1.208	0.150	-1.623	0.198	-0.274	0.110
22	Lack of energy	2.190	0.225	-1.008	0.106	-0.034	0.080
25	Tense	2.109	0.231	-1.567	0.142	-0.511	0.086
26	Easily irritated	1.545	0.169	-1.277	0.141	0.009	0.094
29	Can't do anything anymore	3.122	0.321	-0.572	0.078	0.329	0.074
31	No longer any interest	2.209	0.225	-0.191	0.081	0.805	0.097
32	Can't cope anymore	2.947	0.299	-0.516	0.078	0.475	0.078
36	Can't face it anymore	3.057	0.308	-0.423	0.076	0.541	0.079
37	No longer feel like doing anything	3.483	0.359	-0.640	0.078	0.366	0.073
38	Have difficulty in thinking clearly	1.770	0.181	-0.762	0.104	0.474	0.096
39	Have difficulty in getting to sleep	1.107	0.136	-0.944	0.150	0.427	0.125
41	Easily become emotional	1.760	0.184	-1.075	0.120	0.060	0.088
47	Fleeting images of upsetting event(s)	0.827	0.121	-0.878	0.180	1.177	0.214
48	Do your best to put aside thought about upsetting event(s)	0.992	0.131	-0.443	0.134	1.043	0.174

Notes: α is the discrimination parameter. $ß_1$en $ß_2$ represent the location or difficulty parameters. SE is the standard error of the estimated parameter.

Table 1B. Estimated GRM parameters of the 4DSQ depression scale items

Item #	Item	α	(SE)	$ß_1$	(SE)	$ß_2$	(SE)
28	Everything meaningless	3.499	0.414	-0.018	0.067	0.709	0.079
30	Life is not worth while	6.203	0.995	0.325	0.062	0.941	0.083
33	Would be better off dead	7.377	1.619	0.688	0.071	1.349	0.112
34	Can’t enjoy anything anymore	2.346	0.261	-0.207	0.079	0.741	0.093
35	No escape from situation	2.457	0.281	0.119	0.074	0.828	0.096
46	Think “I wish I were dead”	6.293	1.227	0.777	0.076	1.440	0.128

Table 1C. Estimated GRM parameters of the 4DSQ anxiety scale items

Item #	Item	α	(SE)	$ß_1$	(SE)	$ß_2$	(SE)
18	Sudden fright for no reason	1.369	0.170	0.448	0.105	1.554	0.178
21	Vague feeling of fear	3.886	0.479	-0.303	0.070	0.432	0.069
23	Trembling when with other people	1.874	0.233	0.841	0.102	1.689	0.164
24	Anxiety or panic attacks	2.226	0.255	0.508	0.083	1.400	0.127
27	Frightened	5.527	0.822	-0.140	0.063	0.633	0.070
40	Fear of going out of the house alone	2.032	0.264	0.982	0.106	1.644	0.157
42	Afraid of anything no need for	1.653	0.212	0.872	0.112	1.487	0.160
43	Afraid to travel on buses or other	1.481	0.228	1.536	0.180	2.342	0.283
44	Afraid of becoming embarrassed when with other people	1.323	0.166	0.479	0.108	1.853	0.208
45	Threatened by unknown danger	2.077	0.277	1.108	0.112	1.875	0.177
49	Avoid certain places because they frightened you	1.941	0.272	1.242	0.127	1.880	0.187
50	Repeat some actions a number of times	0.961	0.172	1.689	0.269	2.964	0.474

Table 1D. Estimated GRM parameters of the 4DSQ somatization scale items

Item #	Item	α	(SE)	$ß_1$	(SE)	$ß_2$	(SE)
1	Dizziness or feeling light-headed	1.345	0.161	-0.832	0.128	1.067	0.142
2	Painful muscles	1.431	0.177	-0.622	0.114	0.387	0.105
3	Fainting	1.299	0.337	2.940	0.557	5.217	1.297
4	Neck pain	1.327	0.166	-0.338	0.109	0.776	0.126
5	Back pain	1.480	0.180	-0.316	0.102	0.611	0.111
6	Excessive sweating	0.894	0.133	-0.355	0.143	1.280	0.214
7	Palpitations	1.493	0.180	-0.114	0.097	1.140	0.140
8	Headache	0.988	0.139	-1.398	0.209	0.383	0.133
9	A bloated feeling in the abdomen	1.245	0.158	-0.114	0.108	1.264	0.165
10	Blurred vision or spots in front of eyes	1.268	0.159	-0.039	0.106	1.347	0.170
11	Shortness of breath	1.368	0.178	0.550	0.110	1.695	0.196
12	Nausea or an upset stomach	1.251	0.157	-0.182	0.109	1.260	0.164
13	Pain in the abdomen or stomach area	1.077	0.148	0.118	0.117	1.558	0.212
14	Tingling in the fingers	1.347	0.181	0.712	0.120	1.742	0.207
15	Pressure of tight feeling in the chest	1.699	0.209	0.153	0.090	1.234	0.138
16	Pain in the chest	1.855	0.246	0.840	0.106	1.843	0.186
